# GUIDING FUTURE AUTONOMY-SUPPORTIVE BEHAVIOR BASED ON HISTORICAL KNOWLEDGE OF NURSING THEORIES

**DOI:** 10.1093/geroni/igae098.3993

**Published:** 2024-12-31

**Authors:** Michel Bleijlevens, Melissa Botana Gronek, Stan Vluggen, Esmee Moonen, Aileen van Dijk, Jan Hamers, Judith Meijers

**Affiliations:** Maastricht University, Maastricht, Limburg, Netherlands; Maastricht University, Maastricht, Limburg, Netherlands; Zuyd University of Applied Sciences, Limburg, Netherlands; Maastricht University, Maastricht, Limburg, Netherlands; Maastricht University, Maastricht, Limburg, Netherlands; Maastricht University, Maastricht, Limburg, Netherlands; Maastricht University, Maastricht, Limburg, Netherlands

## Abstract

Autonomy in care provision is central to providing person-centered, ethically sound care that meets the needs and preferences of older people. By stimulating autonomy, nurses can help maintain dignity, enhance quality of life, and promote overall well-being. Nevertheless, there remains a lack of knowledge on what autonomy is and how nurses could show autonomy-supportive behavior. This review examines the concept of autonomy and how nurses can effectively foster it in their ADL practice. In this scoping review, leading nursing theories that shaped the nursing profession and mentioned autonomy or related terms were selected from nursing textbooks, internet searches, and expert consultations. Two researchers independently conducted the screening and analysis using inductive coding. Ultimately, nine theories were included that mentioned related concepts to autonomy such as self-governance. None of the theories explicitly described autonomy or autonomy-supportive behaviors. However, we did find themes related to autonomy such as: being oneself, having a sense of freedom and control, expressing and making choices, and engaging deliberately in actions. Furthermore, six actions were identified to guide nurses in enhancing autonomy: being aware of their own behavior; respecting individual uniqueness; fostering interpersonal connections; facilitating open communication; allowing patients to choose the best course of action; and providing collaborative guidance and assistance. While nursing theories may lack explicit guidelines on autonomy-supportive behaviors, the actions identified in this review offer a practical framework for enhancing autonomy in long-term care. However, further knowledge development in this area is essential in order to stimulate autonomy-supportive behavior.

